# Exploring Signatures of Neurodegeneration in Early-Onset Older-Age Bipolar Disorder and Behavioral Variant Frontotemporal Dementia

**DOI:** 10.3389/fneur.2021.713388

**Published:** 2021-09-03

**Authors:** Francy Cruz-Sanabria, Pablo Alexander Reyes, Cristian Triviño-Martínez, Milena García-García, Claudia Carmassi, Rodrigo Pardo, Diana L. Matallana

**Affiliations:** ^1^Department of Translational Research, New Surgical, and Medical Technologies, University of Pisa, Pisa, Italy; ^2^Neurosciences Research Group, Institute of Genetics, Universidad Nacional de Colombia, Bogotá, Colombia; ^3^Ph.D. Program in Neuroscience, Department of Psychiatry, Pontificia Universidad Javeriana, Bogotá, Colombia; ^4^Radiology Department, Hospital Universitario San Ignacio, Bogotá, Colombia; ^5^Psychiatry Department, School of Medicine, Aging Institute, Pontificia Universidad Javeriana, Bogotá, Colombia; ^6^School of Medicine and Health Sciences, Universidad del Rosario, Bogotá, Colombia; ^7^Department of Clinical and Experimental Medicine, University of Pisa, Pisa, Italy; ^8^Mental Health Department, Hospital Universitario Fundación Santa Fe, Bogotá, Colombia; ^9^Memory and Cognition Clinic, Intellectus, Hospital Universitario San Ignacio, Bogotá, Colombia

**Keywords:** neurodegeneration, structural connectivity, surface-based morphometry, diffusion tensor imaging, neuropsychology, frontotemporal dementia, early-onset older-age bipolar disorder

## Abstract

**Introduction:** Older-age bipolar disorder (OABD) may involve neurocognitive decline and behavioral disturbances that could share features with the behavioral variant of frontotemporal dementia (bvFTD), making the differential diagnosis difficult in cases of suspected dementia.

**Objective:** To compare the neuropsychological profile, brain morphometry, and structural connectivity patterns between patients diagnosed with bvFTD, patients classified as OABD with an early onset of the disease (EO-OABD), and healthy controls (HC).

**Methods:** bvFTD patients (*n* = 25, age: 66 ± 7, female: 64%, disease duration: 6 ± 4 years), EO-OABD patients (*n* = 17, age: 65 ± 9, female: 71%, disease duration: 38 ± 8 years), and HC (*n* = 28, age: 62 ± 7, female: 64%) were evaluated through neuropsychological tests concerning attention, memory, executive function, praxis, and language. Brain morphometry was analyzed through surface-based morphometry (SBM), while structural brain connectivity was assessed through diffusion tensor imaging (DTI).

**Results:** Both bvFTD and EO-OABD patients showed lower performance in neuropsychological tests of attention, verbal fluency, working memory, verbal memory, and praxis than HC. Comparisons between EO-OABD and bvFTD showed differences limited to cognitive flexibility delayed recall and intrusion errors in the memory test. SBM analysis demonstrated that several frontal, temporal, and parietal regions were altered in both bvFTD and EO-OABD compared to HC. In contrast, comparisons between bvFTD and EO-OABD evidenced differences exclusively in the right temporal pole and the left entorhinal cortex. DTI analysis showed alterations in association and projection fibers in both EO-OABD and bvFTD patients compared to HC. Commissural fibers were found to be particularly affected in EO-OABD. The middle cerebellar peduncle and the pontine crossing tract were exclusively altered in bvFTD. There were no significant differences in DTI analysis between EO-OABD and bvFTD.

**Discussion:** EO-OABD and bvFTD may share an overlap in cognitive, brain morphometry, and structural connectivity profiles that could reflect common underlying mechanisms, even though the etiology of each disease can be different and multifactorial.

## Introduction

Bipolar disorder (BD) is a chronic psychiatric disease associated with excitotoxicity and neuroinflammation processes that may contribute, among other factors, to accelerate normal aging mechanisms (1, 2); therefore, its progression as a neurodegenerative disorder has been explored (3, 4). Patients with BD frequently suffer from cognitive deficits that may persist during periods of euthymia (5–7). However, cognitive impairment in BD is heterogeneous (8, 9); it may remain stable over time (10–13) or may have a progressive course (14) that could be accompanied by progressive loss of gray matter (15) and disability (9, 16). Indeed, a history of BD may significantly increase the risk of dementia in older adults (17); nonetheless, a differential diagnosis regarding the type of dementia may represent a challenge. The existence of a specific dementia derived from the evolution of BD and characterized by a different profile from typical neurodegenerative conditions has been proposed (18). However, other authors have suggested that elderly BD patients may progress to neurodegenerative disorders that could fall into syndromes belonging to the frontotemporal lobar degeneration (FTLD) spectrum (19, 20).

Patients with BD who are around the sixth decade of their lives are defined as older-age bipolar disorder (OABD). It represents a heterogeneous group that includes both patients with an early onset of the disease (EOBD), referring to those patients who have their first manic/hypomanic episode at <50 years old, and patients with a late onset of the disease (LOBD), referring to those patients who have their first manic/hypomanic episode aged >50 years. Nonetheless, a cut-point of 40 years has been also proposed to discriminate between EOBD and LOBD, and a cut-point of >50 years old has been proposed as the age to consider patients as belonging to the group of OABD given the reduced lifespan and the high medical burden reported in BD (21). The link between OABD and FTLD, particularly with the behavioral variant of frontotemporal dementia (bvFTD), is complex and heterogeneous. On the one hand, clinical reports have described that early-onset OABD patients may develop progressive cognitive impairment, particularly in executive functions (EF), together with behavioral changes and predominant atrophy in frontotemporal regions, constituting cases in which a differential diagnosis regarding frontotemporal dementia (FTD) is challenging (19, 20, 22–25). However, the link between bvFTD and BD involves also late-onset OABD, with patients who initiate mood and behavioral alterations at ≥50 years old and that may exhibit similar symptoms to those observed in bvFTD (26–28). Likewise, in bvFTD the probability of receiving an erroneous diagnosis of psychiatric disease such as BD is significantly higher than in other neurodegenerative disorders (29). A retrospective study based on the psychiatric history of 137 patients with bvFTD found that 10.2% of patients had a previous history of BD, which is significantly higher than the prevalence in the general population (2.6%) (30). Moreover, a shared genetic pre-disposition between BD and FTLD has been considered due to evidence of mutations in the C9ORF72 gene in a BD patient that evolve to FTD (22) and in a family that included both BD and DFT diagnosis (31). Also, mutations in the progranulin gene in patients with FTLD and premorbid bipolar spectrum disorders (19) and in a case of late-onset BD that develop bvFTD (32) as well as lower progranulin plasma levels reported in BD compared to healthy controls (HC) (33, 34) point to common genetic pre-disposing factors. In this context, it has been suggested that BD could constitute a long-standing pre-clinical phase that precedes some FTLD disorders (19). Although the presence of common molecular mechanisms underlying both BD and FTD has been extensively explored (35), whether BD in particular may progress to dementia associated with bvFTD remains to be elucidated.

In addition to common clinical profiles regarding cognitive dysfunction, common neuroanatomical changes have also been described in prefrontal regions, anterior temporal lobes, and limbic structures in both BD (4, 36, 37) and bvFTD (38, 39), with deficiencies in functional and structural connectivity that may particularly involve frontal networks (40, 41). However, comparative studies between OABD and bvFTD are scarce. A previous study in which OABD patients were compared with bvFTD patients (42) found that although both clinical conditions exhibited alterations in EF, in bvFTD cognitive deficits and atrophy in frontal, temporal, and parietal regions were greater; moreover, the morphometric profile was associated with EF and social cognitive performance only in the bvFTD group. Likewise, a recent study combining magnetic resonance imaging (MRI) and positron emission tomography (PET) techniques report that although both elderly BD and bvFTD patients showed prefrontal cortex (PFC) reduction, the first group showed greater alteration in the ventrolateral prefrontal cortex (VLPFC), while the latter group showed deeper alteration in the dorsolateral prefrontal cortex (DLPFC); moreover, bvFTD patients showed more extensive alterations in limbic regions than elderly BD and particular volumetric and metabolic reductions in regions within the temporo-parietal network (43). These results suggest differential characteristics between BD and bvFTD that deserve to be further explored. Since structural and functional connectivity may change due to reorganization derived from the evolution of neurodegenerative and psychiatric diseases, it is relevant to study connectivity features in BD and bvFTD.

On the one hand, white matter (WM) abnormalities are frequent in BD (44, 45), in which alterations in oligodendrocytes and myelination constitute possible underlying disease mechanisms (41) and may significantly affect connectivity patterns. Indeed, BD does not appear to be correlated with changes in specific brain areas. Still, it possibly corresponds to disruption in several brain networks, which is reflected by a large constellation of symptoms that characterize this clinical condition, including emotional, cognitive, behavioral, autonomic, neuroendocrine, immune, and circadian disturbances (46). On the other hand, in bvFTD, it has been reported that changes in gray matter tend to occur together with WM disruptions (47–49) and that the alterations in multiple cognitive functions observed in bvFTD may result as a consequence of the poor integration of networks which reduce the ability to combine specialized information from distributed brain regions (50). Thus, even when both clinical conditions have been described as “connectivity disorders” (41, 51, 52), so far, no study has compared structural connectivity features between OABD and bvFTD. We conducted this investigation to identify neurocognitive and neuroimaging markers based on WM integrity measured through tract-based spatial statistics, cortical thickness explored through surface-based morphometry (SBM), and neuropsychological profiles in patients with an early-onset older-age bipolar disorder (EO-OABD) compared with patients diagnosed with bvFTD and HC.

## Methods

### Participants

Overall, 25 patients with a diagnosis of bvFTD were consecutively enrolled for the present study. The diagnosis was determined through consensus by a multidisciplinary group of specialists (neurology, geriatrics, psychiatry, and neuropsychology) at the Memory Clinic of the Hospital Universitario San Ignacio (Bogotá, Colombia) based on the guidelines developed by an international consortium for the diagnosis of FTD (53). Since histopathological evidence of FTLD was not available and the presence of a known pathogenic mutation was not tested, a definitive diagnosis of bvFTD was not established. However, all patients fulfilled the diagnosis of *Probable bvFTD*, so that they met clinical criteria for possible bvFTD and showed significant functional impairment, and imaging results showed frontal and/or anterior temporal atrophy on MRI. We also included 17 patients diagnosed with BD attending the Memory Clinic, who reported a history of more than 20 years of evolution of the psychiatric disease. The inclusion criteria for the BD group consisted of a Diagnostic and Statistical Manual of Mental Disorder (DSM)-5 diagnosis of BD (I–II), euthymic phase confirmed by a total score <7 in the Hamilton Depression Rating Scale (HDRS) (54), and the absence of manic symptoms based on the psychiatric interview. The psychiatric evaluation was performed by a psychiatrist expert on psychogeriatrics using both a semi-structured interview and complementary scales that included, besides the HDRS, the Cornell Scale for Depression in Dementia (55) and the Columbia University Scale for Psychopathology in Alzheimer's Disease, which allows evaluating symptoms of psychosis, behavioral disturbance, and depression (56). In this way, through the overall evaluation, information about symptoms such as agitation, aggression, irritability, thought disturbance, and changes in sleeping and eating patterns was collected, which allowed discarding a manic/hypomanic episode. Exclusion criteria for both clinical groups include visual and hearing impairments, severe alteration of mobility, delirium, absence of caregiver or informant, and significant cerebrovascular disease. HC were enrolled through a public call. Inclusion criteria for HC involved a negative history of psychiatric or neurologic disorders, no complaints of recent cognitive or behavioral changes, and a Montreal Cognitive Assessment test (MoCA) (57) score higher than 24. All eligible subjects were asked to provide written informed consent after receiving a complete description of the study and having an opportunity to ask questions before joining the study. This study was approved by the Ethics Committee of the Hospital Universitario San Ignacio and the Pontificia Universidad Javeriana.

### Neuropsychological Assessment

Cognitive functions concerning attention, memory, EF, praxis, and language were evaluated through the Symbol Digit Modalities Test (SDMT) (58) and the Grober–Buschke test for explicit verbal memory, which evaluates immediate and delayed recovery using a paradigm of Free and Cued Selective Reminding Test (FCSRT) (59), the Rey–Osterrieth Complex Figure (ROCF) (60), the Semantic and Phonological verbal fluency (61), the Wisconsin Card Sorting Test (WCST) (62), and the Institute of Cognitive Neurology (INECO) Frontal Screening (IFS), which measures different aspects of EFs such as motor programming, motor and verbal inhibitory control, working memory, and abstraction capacity (63). In addition, the MoCA (57) test was used to establish a global cognitive profile.

### Image Acquisition and Processing

#### MRI Data Acquisition

The structural MRI scans were obtained on a 3T MR Scanner (Philips Achieva). The T1-weighted images of the whole brain (220 sagittal slices, 0.5 × 0.5 × 0.5 mm) were acquired with a gradient-echo sequence: repetition time = 7.7 ms, echo time = 3.7 ms, field of view = 256 × 256.

#### Data Processing

The SBM analysis was performed with the CAT12 Toolbox (http://dbm.neuro.uni-jena.de/cat/) in SPM12 (Wellcome Center for Neuroimaging, http://www.fil.ion.ucl.ac.uk/spm/) (64), implemented on MATLAB R2017b software (MathWorks, Natick, MA, USA). The CAT12 Toolbox contains a processing pipeline for SBM, which includes an established novel algorithm for extracting the cortical surface (65), thus allowing the computation of multiple morphometric parameters (including cortical surface and gyrification index).

In order to estimate WM distances, the T1-weighted images were subjected to tissue segmentation. Local maxima were then projected to other gray matter voxels by using a neighbor relationship described by the WM distance (65). These values equal cortical surface. This projection-based method also includes partial volume correction, sulcal blurring, and sulcal asymmetries without sulcus reconstruction. A topological correction was performed through an approach based on spherical harmonics. For inter-patient analyses, an algorithm for spherical mapping of the cortical surface was included (66). An adapted volume-based diffeomorphic anatomical registration through the exponentiated lie algebra (DARTEL) algorithm was then applied to the surface for spherical registration (67).

In addition to cortical surface analysis, we extracted the local gyrification index based on the absolute mean curvature (68). Central cortical surfaces were created for both hemispheres separately. Finally, all scans were re-sampled and smoothed with a Gaussian kernel of 15 mm full width at half-maximum (FWHM) for the cortical surface and with a 20 mm FWHM for the gyrification index.

### Statistical Analysis

Significant differences between groups were evaluated based on *post-hoc* comparisons (*p* < 0.05), following a one-way analysis of variance (ANOVA) significant at *p* < 0.05 or Kruskal–Wallis for variables with no normal distribution. We used R software (version. 3.5.0) for the statistical analysis of clinical and neuropsychological features.

Regarding the SBM analysis, we applied the general linear models to the individual maps and then carried out a multiple regression analysis on the individual cortical surface and gyrification index maps. Age was considered as a nuisance factor to correct for age differences. For the multiple regression analysis, threshold-free cluster enhancement (TFCE) was used (69) after correcting for multiple comparisons across space using false discovery rate (FDR) correction. The anatomical locations of the significant clusters were determined with reference to the multi-modal analyses of magnetic resonance images from the Human Connectome Project (HCP) (70).

A *post-hoc* analysis was conducted to test the correlation between cortical thickness and cognitive performance, with a particular interest in the cognitive domains that showed significant differences between BD and bvFTD. The average thickness value of a series of regions of interest (ROIs) was automatically produced by the CAT12 Toolbox (71). Correlations between average thickness and clinical measurements, including disease duration and neuropsychological tests of memory and EF, were analyzed using a Spearman test significant at *p* < 0.05.

## Results

### Sample Description

A sample of 25 patients with diagnosis of bvFTD (age: 66 ± 7, females: 64%), 17 patients with diagnosis of BD (type I: *n* = 14, type II: *n* = 3), in euthymia (age: 65 ± 9, females: 71%), as well as 28 age- and education-matched HC (age: 62 ± 7, females: 64%) were included in this study. Significant differences (*p* < 0.001) were found between the clinical groups regarding the *onset age* and the *disease duration*, being significantly longer in the BD group than in the bvFTD group. The onset age of the neurodegenerative disease in the bvFTD group was 59 ± 7 (median 59, range: 41–74) with a disease duration of 6 ± 4 years (median 7, range: 1–16). In the BD group, the onset age of the psychiatric disease was 27 ± 7.5 (median 27, range: 17–41), and the disease duration was 38 ± 8 years (median 36, range: 23–51). Based upon the hierarchical terminology proposed by the International Society of Bipolar Disorders (ISBD) task force on OABD (21), our sample may be classified as OABD since the overall sample aged ≥50 years old. Moreover, our sample may be classified as early-onset BD (EOBD), since the first manic/hypomanic episode was presented at <40 years old in the 94% of cases [Only one patient reported his first manic episode at 41 years old: close to the cut-point proposed by the ISBD (<40 years old) and among the range generally considered as early-onset (<50 years old)]. Therefore, our sample was classified as early-onset Older Age Bipolar Disorder (EO-OABD). A history of mixed episodes was identified in three patients (17%), and a baseline cyclothymic disorder was described in one patient (5.8%). Psychotropic drugs administered to the EO-OABD group at the moment of the evaluation included: mood stabilizers such as antiepileptics (64.7%) and lithium (29.4%), antipsychotic drugs (88.2%), antidepressants (29.4%), benzodiazepines (BZD) (35.3%), and hypnotics/sedatives no BZD (17.6%). In the bvFTD group, psychotropic drugs were also present, including antidepressants (40%), BZD (40%), and antipsychotics (4%). Moreover, one bvFTD patient was being treated with lithium as a mood stabilizer. These drugs in the bvFTD group were administered to treat behavioral and mood changes produced in the context of the neurodegenerative disease. Only one bvFTD patient has a personal history of a depressive episode reactive to a stressful event and not related to the actual disease. Comparisons of the comorbidities and other clinical data between the group of patients showed significant differences regarding the familial history of psychiatric disease (*p* < 0.001), where EO-OABD patients showed a higher prevalence than bvFTD patients (94 vs. 32%, respectively). Significant differences were also found concerning the history of alcohol consumption, being more prevalent in EO-OABD than in bvFTD (35.3 vs. 4%, respectively). Risk factors for vascular disease showed differences regarding the history of diabetes mellitus, with a higher prevalence among EO-OABD than in bvFTD (23 vs. 4%, respectively). As expected, patients also differ in the history of psychotic symptoms, being more prevalent in EO-OABD than in bvFTD (52.9 vs. 12%, respectively). Demographic and clinical data are summarized in Tables 1, 2.

**Table 1:** Demographic data and neuropsychological profiles.

	**EO-OABD** ***n*** **=** **17**		**bvFTD** ***n*** **=** **25**		**HC** ***n*** **=** **28**		
	**Mean (SD)**	**Median (min–max)**	**Mean (SD)**	**Median (min–max)**	**Mean (SD)**	**Median (min–max)**	***p*-value**
Age	65 ± 9	64 (54–81)	66 ± 7	65 (51–78)	62 ± 7	61 (50–80)	0.120^d^
Years of education	14 ± 6	16 (2–22)	13 ± 5	16 (4–20)	15 ± 5	16 (5–20)	0.271^e^
Onset age	27 ± 7.5	27 (17–41)	59 ± 7	59 (41–74)	-	-	<0.001
Disease duration (in years)	38 ± 8	36 (23–51)	6 ± 4	7 (1–16)	-	-	<0.001
FCSRT Free recall total^a^^,^ ^b^	21 ± 7	23 (10–31)	14 ± 9	12 (2–33)	28 ± 6	29.5 (13–37)	<0.001^e^
FCSRT Recall total^a^^,^ ^b^	40 ± 8	43.5 (22–48)	32 ± 13	32 (6–48)	46 ± 2	47 (40–48)	<0.001^e^
FCSRT Delayed recall^a^^,^ ^b^^,^ ^c^	8 ± 3	7 (2–15)	5 ± 4	4 (0–13)	11 ± 2	11 (6–115)	<0.001^d^
FCSRT Delayed recall total^a^^,^ ^b^^,^ ^c^	14 ± 3	14 (6–16)	10 ± 6	11 (0–16)	16 ± 1	16 (13–16)	<0.001^e^
FCSRT Intrusion errors^a^^,^ ^c^	3.4 ± 4.5	1 (0–13)	14.5 ± 16.7	6 (0–53)	1.1 ± 1.6	0 (0–6)	<0.001^e^
IFS total score^a^^,^ ^b^	16.6 ± 6.5	18 (5–25)	12.6 ± 6.0	12.5 (0–24)	22.7 ± 3.2	22.7 (13–27)	<0.001^e^
WorkMem IFS^a^^,^ ^b^	3.0 ± 1.8	3 (2–7)	3.6 ± 1.3	4 (0–6)	5.6 ± 1.6	5.5 (3–9)	<0.001^e^
WCST Conceptualization^a^^,^ ^b^^,^ ^c^	55 ± 26	44 (21–92)	43 ± 24	44 (8–86)	79 ± 16	85 (48–100)	<0.001^e^
WCST Correct^a^	28 ± 11	32 (10–42)	26 ± 10	25 (7–41)	35 ± 5	36 (26–43)	0.003^e^
WCST Categories^a^	4 ± 2	4 (1–6)	3 ± 2	2 (0–6)	5 ± 1	6 (2–6)	<0.001^e^
WCST Perseverations^a^	20.4 ± 19	11 (2–63)	22.8 ± 22	18 (2–83)	8.8 ± 8	7 (0–29)	0.010^d^
WCST Attentional Errors^a^	1 ± 1	0 (0–3)	2 ± 3	1 (0–9)	0 ± 1	0 (0–3)	0.011^e^
SDMT^a^^,^ ^b^	34 ± 21	32 (3–66)	28 ± 16	24 (3–60)	51 ± 16	52 (20–81)	<0.001^d^
Semantic VF^a^^,^ ^b^	12.7 ± 5	14 (6–21)	11.8 ± 5.3	12 (3–23)	17.1 ± 2.7	16.5 (12–22)	<0.001^d^
Phonological VF^a^^,^ ^b^	11.7 ± 5.7	13.5 (2–22)	11.3 ± 4.9	10.5 (5–21)	15.5 ± 4.5	15.2 (7–24)	0.005^d^
ROCF Correction^a^^,^ ^b^	23.7 ± 11	27 (9–36)	22.7 ± 11.5	26 (0–36)	34 ± 2	34 (30–36)	<0.001^e^
ROCF Time (seg)^b^	258 ± 54	285 (180–300)	216 ± 88.4	240 (77–300)	163 ± 66.6	156 (60–300)	0.015^e^

*Results are presented in mean (SD) and median (range). Comparisons between groups were performed through one-way ANOVA for normal distribution (Shapiro–Wilk test, p > 0.05) or Kruskal–Wallis analysis for variables with no normal distribution. Comparisons including only clinical groups (BD vs. bvFTD), as for disease duration and Onset age variables, were performed through Student's t-test. Significant differences were considered at p < 0.05*.

a *The post-hoc results are presented as significant differences between HC and bvFTD*.

b *The post-hoc results are presented as significant differences between HC and BD*.

c *The post-hoc results are presented as significant differences between BD and bvFTD*.

d *ANOVA, post-hoc: Bonferroni test*.

e *Kruskal–Wallis test*.

*EO-OABD, early-onset Older Age Bipolar disorder; bvFTD, behavioral variant frontotemporal dementia; HC, healthy controls; SD, standard deviation; FCSRT, Free and Cued Selective Reminding Test; IFS, INECO Frontal Screening; WorkMem IFS, Subtest on working memory, INECO frontal screening; INECO, Institute of Cognitive Neurology; WCST, Wisconsin Card Sorting Test; SDMT, Symbol Digit Modalities Test; VF, verbal fluency; ROCF, Rey–Osterrieth Complex Figure; ANOVA, analysis of variance*.

**Table 2:** Comorbidities and psychotropic medications.

	**EO-OABD (*n* = 17)**	**bvFTD (*n* = 25)**	***p*-value**
**Clinical data and comorbidities**	***N*** **(%)**	***N*** **(%)**	
Psychotic symptoms	9 (52.9%)	3 (12%)	0.006
Familial history of dementia	6 (35.3%)	11 (44%)	0.75
Familial history of psychiatric disease	16 (94%)	8 (32%)	<0.001
Cigarette consumption	9 (52%)	11 (44%)	0.74
Alcohol consumption	6 (35.3%)	1 (4%)	0.01
Hypertension	5 (29.4%)	7 (28%)	>0.999
Diabetes mellitus	4 (23%)	1 (4%)	0.07
Hyperlipidemia	3 (12%)	2 (11%)	1.000
Coronary artery disease	3 (17%)	1 (4%)	0.173
**Psychotropic medications**
Antidepressant	5 (29.4%)	10 (40%)	0.531
Mood stabilizers (antiepileptic)	11(64.7%)	0	<0.001
Lithium	5 (29.4%)	1 (4%)	0.032
Benzodiazepines (BZD)	6 (35.3%)	10 (40%)	>0.999
Antipsychotics	15 (88.2%)	1 (4%)	<0.001
Hypnotics/sedatives (no BZD)	3 (17.6%)	0	0.059

*Categorical variables were compared through the Fisher test. Significant values were considered at p < 0.05*.

*EO-OABD, early-onset Older Age Bipolar disorder; bvFTD, behavioral variant frontotemporal dementia*.

### Neuropsychological Profile

Between-group comparisons and *post-hoc* analysis (Table 1) revealed that in the cognitive screening test (MoCA), the performance was significantly lower in the bvFTD (18.5 ± 6.2, *p* < 0.001) and EO-OABD (22.1 ± 4.8, *p* < 0.05) groups when compared to HC (26.3 ± 2.5), while no significant differences were found between EO-OABD and bvFTD. Similarly, in memory variables of immediate free and cued recovery (*FCSRT Free recall total* and *FCSRT Recall total*), lower performances were observed in bvFTD (*p* < 0.001) and EO-OABD (*p* < 0.05) when compared to HC, while no differences were found between EO-OABD and bvFTD. In memory variables of delayed free and cued recovery (*FCSRT Delayed recall* and *FCSRT Delayed recall total*), the performance was significantly lower in bvFTD (*p* < 0.001) and EO-OABD (*p* < 0.05) when compared with HC; in addition, in the bvFTD group, lower scores were found than in EO-OABD (*p* < 0.05). A significantly greater number of intrusion errors—a variable that quantifies the number of not related information that emerged during recall processes—was observed in bvFTD when compared with both HC and EO-OABD (*p* < 0.001). In tests evaluating EF, both bvFTD and EO-OABD patients showed lower performances than HC, including the *IFS total score* (*p* < 0.001 and *p* < 0.05, respectively), the *working memory test* (*p* < 0.001 and *p* < 0.05, respectively), *WCST conceptualization* (*p* < 0.001 and *p* < 0.05, respectively), and the *phonological verbal fluency* (*p* < 0.05 in both cases). Among the EF tests described, only the variable *WCST conceptualization* showed significant differences between bvFTD and EO-OABD, with a lower performance in the bvFTD group compared to EO-OABD (*p* < 0.001). In other variables derived from WCST, performance was significantly lower only in bvFTD when compared to HC, including the number of correct responses (*p* = 0.003), categories completed (*p* < 0.001), perseveration (*p* = 0.010), and attentional errors (*p* = 0.011). Regarding other cognitive processes, significantly lower performances were found in both bvFTD and EO-OABD patients when compared to HC in tests evaluating attention through *SDMT* (*p* < 0.001), language evaluated through the *semantic verbal fluency test* (*p* < 0.001), and praxis as evaluated through the *ROCF* (*p* < 0.001), while no significant differences were found in these variables between EO-OABD and bvFTD. Finally, the EO-OABD group showed lower processing speed, as measured through *ROCF time*, compared to HC (*p* = 0.015).

### Brain Morphometry

**Figure 1:**
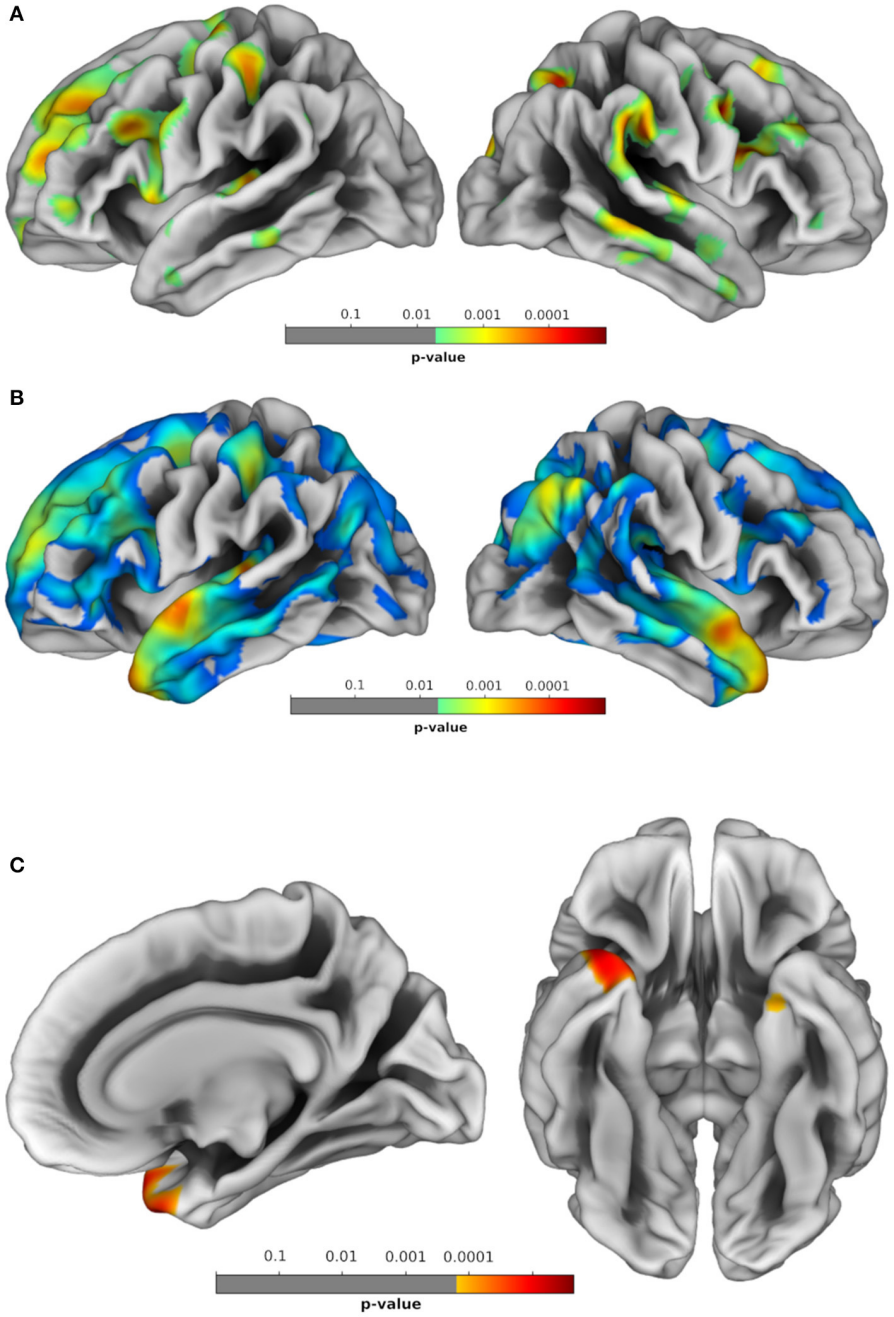
Brain regions showing significant statistical differences between groups in morphometric profiles. **(A)** HC vs. EO-OABD; **(B)** HC vs. bvFTD; **(C)** EO-OABD vs. bvFTD. False discovery rate (FDR) correction for multiple comparisons was applied with cluster significance of *p* < 0.05 and cluster size >30. HC, healthy controls; EO-OABD, early-onset older-age bipolar disorder; bvFTD, behavioral variant of frontotemporal dementia.

Differences in cortical surface, as evaluated through SBM with FDR correction (*p* < 0.05), showed that compared to HC, EO-OABD patients exhibited decreased surface in cortical regions of the right hemisphere (R) belonging to the frontal lobe (rostral middle frontal, caudal middle frontal, pars opercularis, pars triangularis, superior frontal, and pre-central), temporal lobe (superior temporal, transverse temporal, middle temporal, and inferior temporal), parietal lobe (supramarginal and superior parietal), and occipital lobe (lateral occipital and cuneus). Likewise, in the left hemisphere (L), decreased cortical surface was observed in the frontal lobe (rostral middle frontal, superior frontal, caudal middle frontal, pars opercularis, pars triangularis, pars orbitalis, frontal pole, pre-central, and paracentral), temporal lobe (superior temporal and transverse temporal), parietal lobe (post-central, supramarginal, paracentral, and precuneus), and occipital lobe (cuneus). See Figure 1A and Table 3.

**Table 3:** Regional brain differences in morphometric profiles between HC and EO-OABD patients.

**Cluster equivk**	**Peak *p* (FDR-corr)**	**MNI Coordinates**			
		**x**	**y**	**z**	
772	0.032	42	1	38	Precentral, rostral middle frontal, pars opercularis, caudal middle frontal R
281	0.032	27	−64	53	Superior parietal R
291	0.032	−29	−14	62	Precentral, superior frontal, caudal middle frontal L
989	0.032	53	−30	32	Supramarginal, superior temporal, transverse temporal R
63	0.032	−40	32	−2	Pars triangularis L
794	0.032	−40	19	28	Pars opercularis, precentral, caudal middle frontal, rostral middle frontal L
343	0.032	−47	−26	47	Postcentral, supramarginal L
177	0.032	−48	−24	10	Superior temporal, transverse temporal L
885	0.032	−19	42	33	Rostral middle frontal, superior frontal, caudal middle frontal L
130	0.032	−15	−40	53	Paracentral, precuneus L
90	0.032	13	−90	24	Superior parietal, cuneus, lateral occipital R
207	0.032	68	−33	−6	Middle temporal, inferior temporal R
86	0.032	42	32	−1	Pars triangularis R
114	0.032	24	22	55	Superior frontal R
122	0.032	24	−8	56	Precentral, superior frontal R
42	0.032	−12	59	−15	Rostral middle frontal, frontal pole L
203	0.032	−5	−10	61	Superior frontal, paracentral, precentral L
62	0.033	−44	44	−4	Rostral middle frontal, pars triangularis, pars orbitalis L
54	0.034	−12	−65	18	Cuneus, precuneus L
52	0.035	57	7	−29	Middle temporal R
51	0.039	48	2	−23	Superior temporal R

*HC, healthy controls; EO-OABD, early-onset Older Age Bipolar disorder; FDR, false discovery rate; MNI, Montreal Neurological Institute; R, right hemisphere; L, left hemisphere*.

Significant reduction in the cortical surface was found in bvFTD when compared to HC in R cortical regions belonging to the frontal lobe (pars opercularis, pars orbitalis, pars triangularis, rostral middle frontal, caudal middle frontal, superior frontal, and pre-central), temporal lobe (superior temporal), and parietal lobe (inferior parietal, precuneus, superior parietal, and post-central), as well as in L cortical regions belonging to the frontal lobe (superior frontal and rostral middle frontal), temporal lobe (fusiform and parahippocampal), parietal lobe (precuneus, supramarginal, post-central, and superior parietal), and occipital lobe (lingual), as well as in posterior and isthmus regions of the cingulate. See Figure 1B and Table 4.

**Table 4:** Regional brain differences in morphometric profiles between HC and bvFTD patients.

**Cluster equiv *k***	**Peak *p* (FDR-corr)**	**MNI Coordinates**			
		**x**	**y**	**z**	
9,702	0.001	22	10	−36	Superior temporal, inferior parietal, precuneus, superior parietal R
11,705	0.001	−30	16	−41	Superior frontal, precuneus, rostral middle frontal L
318	0.001	−2	−23	32	Posterior cingulate, isthmus cingulate L
1,135	0.001	−41	−28	43	Supramarginal, postcentral, superior parietal L
1,177	0.002	44	9	30	Precentral, pars opercularis, rostral middle frontal, pars triangularis, caudal middle frontal R
1,362	0.002	25	−7	61	Superior frontal, precentral, caudal middle frontal R
313	0.009	−25	−72	−9	Fusiform, lingual L
82	0.018	32	−43	45	Superior parietal R
125	0.02	37	−27	60	Postcentral, precentral R
58	0.026	−31	−43	−6	Lingual, fusiform, parahippocampal L
63	0.033	28	−35	54	Postcentral R
42	0.041	49	39	−11	Rostral middle frontal, pars orbitalis, pars triangularis R

*HC, healthy controls; bvFTD, behavioral variant frontotemporal dementia; FDR, false discovery rate; MNI, Montreal Neurological Institute; R, right hemisphere; L, left hemisphere*.

Between EO-OABD and bvFTD, significant differences in cortical surface were found in the right temporal pole and the left entorhinal cortex, where the bvFTD group showed a more substantial decrease. See Figure 1C and Table 5.

**Table 5:** Regional brain differences in morphometric profiles between EO-OABD and bvFTD patients.

**Cluster**	**Peak**	**MNI Coordinates**			
**Equivk**	***p* (FDR-corr)**	**x**	**y**	**z**	
283	0.02	38	17	−42	Temporal pole R
119	0.04	−26	−5	−36	Entorhinal L

*EO-OABD, early-onset Older Age Bipolar disorder; bvFTD, behavioral variant frontotemporal dementia; FDR, false discovery rate; MNI, Montreal Neurological Institute; R, right hemisphere; L, left hemisphere*.

### Structural Connectivity

Comparisons regarding fractional anisotropy (FA), a measure of WM integrity, using a threshold of *p* < 0.05 (FDR corrected), did not show significant differences between groups in any of the contrasts performed (HC > bvFTD, HC > EO-OABD, EO-OABD > bvFTD, bvFTD > EO-OABD). However, using a less restrictive threshold (*p* < 0.001, uncorrected), some differences emerged for comparisons between HC and bvFTD, as well as between HC and EO-OABD.

**Figure 2:**
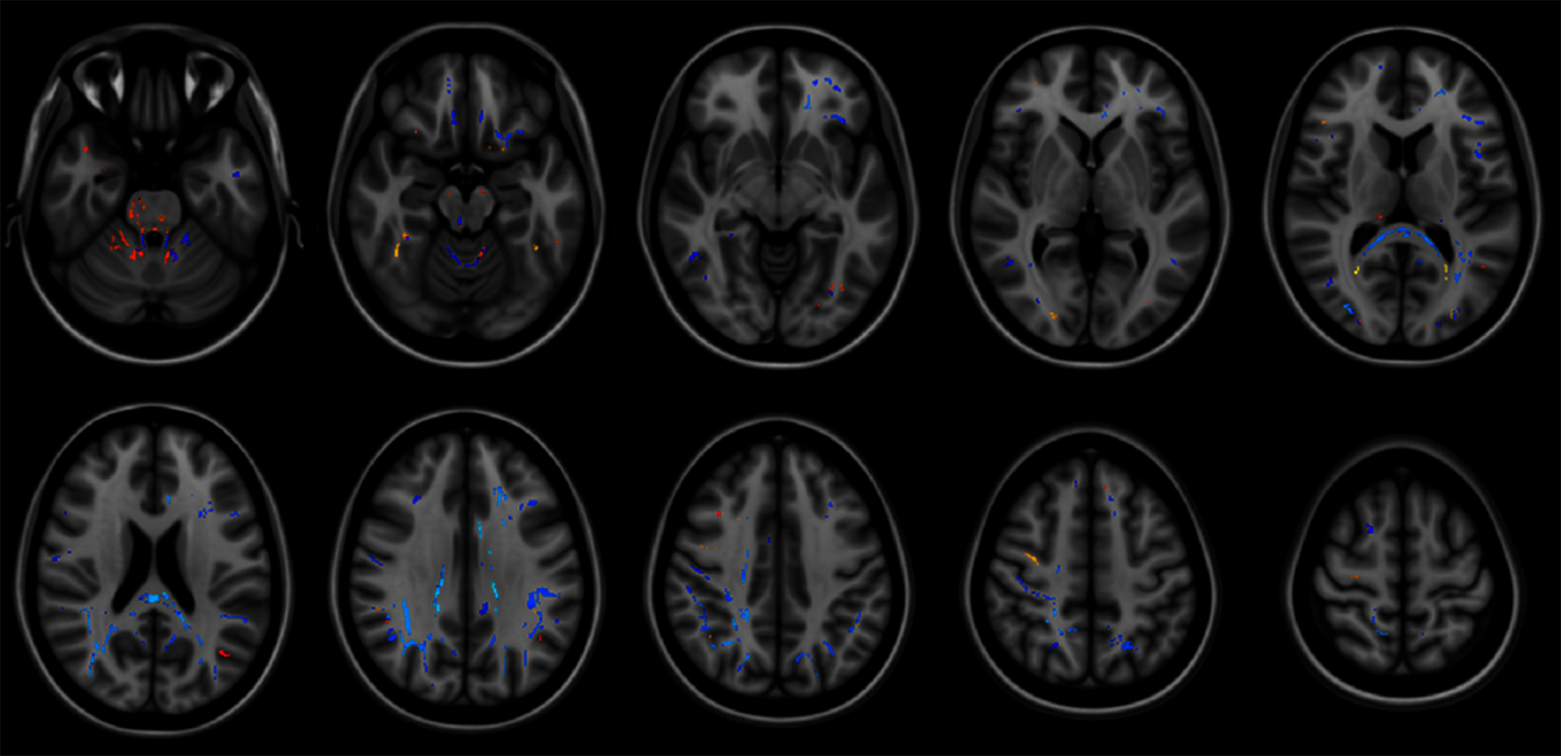
Brain regions showing significant statistical differences between groups in structural connectivity as measured by fractional anisotropy (FA). Correction at *p* = 0.01. Findings in HC vs. FTD are shown in yellow-red. Findings in HC vs. EO-OABD are shown in blue. The background images on each panel are study-specific templates in MNI space. The right side of the images represents the left side of the brain. HC, healthy controls; FTD, frontotemporal dementia; EO-OABD, early-onset older-age bipolar disorder; MNI, Montreal Neurological Institute.

In the EO-OABD group, when compared to HC patients, FA differences were found in commissural fibers such as the body of corpus callosum (L/R) and the forceps minor and major; in association fibers including the superior longitudinal fasciculus (SLF) (L/R), uncinate fasciculus (L), inferior fronto-occipital fasciculus (IFOF) (L), cingulum (L/R), and inferior longitudinal fasciculus (ILF) (L/R); in projection fibers such as the anterior thalamic radiation (ATR) (L/R), anterior corona radiata (L), corticospinal tract (CST) (R), and posterior thalamic radiation (PTR), as well as in WM of the superior cerebellar peduncle (L/R), cerebellum (L/R), and adjacent to the lateral occipital cortex superior division (L/R), angular gyrus (L), precuneus cortex (L), frontal orbital cortex (L), middle frontal gyrus (L), post-central gyrus (R), frontal medial cortex (R), superior frontal gyrus (L), planum temporale (L), subcallosal cortex (L), lateral occipital cortex inferior division (R), precuneus cortex (R), and middle temporal gyrus (L). See Table 6 and Figure 2.

**Table 6:** Regional brain differences in structural connectivity as measured by FA between HC and EO-OABD patients at *p* < 0.01, corrected for multiple comparisons.

**Cluster equivk**	**Peak**	**MNI coordinates**			**Region with FA differences**
	***p* (unc)**	**x**	**y**	**z**	
5,775	0.008	−9	−22	27	Body of corpus callosum L
967	0.005	−40	−32	32	Superior longitudinal fasciculus L
564	0.004	5	−55	−17	WM in right cerebellum
389	0.004	−21	−60	47	WM adjacent to lateral occipital cortex, superior division L, and angular gyrus L
363	0.010	−31	32	−6	Uncinate fasciculus L, inferior fronto-occipital fasciculus L
347	0.005	−29	22	39	WM adjacent to middle frontal gyrus L
254	0.010	−10	7	26	Body of corpus callosum L
253	0.007	30	−31	42	Superior longitudinal fasciculus R
241	0.001	42	−23	50	WM adjacent to postcentral gyrus R
234	0.009	−16	−56	31	Cingulum (cingulate gyrus) L
208	0.007	−43	31	5	Inferior fronto-occipital fasciculus L
166	0.006	−11	−68	41	WM adjacent to precuneus cortex L
153	0.009	−24	−68	28	WM adjacent to the inferior longitudinal fasciculus L
148	0.006	−33	−77	15	Inferior longitudinal fasciculus L
141	0.009	−27	44	−4	Inferior fronto-occipital fasciculus L
133	0.008	49	−52	−1	Adjacent to the superior longitudinal fasciculus R
122	0.006	8	45	−20	WM adjacent to frontal medial cortex R
120	0.010	−11	15	55	WM adjacent to superior frontal gyrus L
110	0.010	−18	42	8	Anterior corona radiata L, forceps minor L
94	0.007	−51	−32	7	WM adjacent to the planum temporale L
92	0.005	−7	30	−22	Cingulum (cingulate gyrus) L
91	0.005	12	−40	67	Corticospinal tract R
91	0.008	7	−42	−27	Anterior thalamic radiation R, superior cerebellar peduncle R
89	0.005	−20	29	27	Anterior thalamic radiation L
87	0.007	38	−70	8	Adjacent to the inferior longitudinal fasciculus
81	0.010	−27	−62	17	Posterior thalamic radiation, forceps major, inferior longitudinal fasciculus L
71	0.009	11	−64	36	WM adjacent to precuneus cortex R
70	0.005	−5	26	−1	Forceps minor, genu of corpus callosum L
69	0.008	−11	−38	31	Cingulum (cingulate gyrus) L
68	0.008	−51	−56	7	WM adjacent to middle temporal gyrus, temporo-occipital part L
62	0.009	−17	38	−4	Anterior corona radiata L, forceps minor, uncinate fasciculus L
57	0.008	−29	−58	15	Posterior thalamic radiation L, forceps major, inferior fronto-occipital fasciculus L

*FA, fractional anisotropy; HC, healthy controls; EO-OABD, early-onset Older Age Bipolar disorder; MNI, Montreal Neurological Institute; L, left hemisphere; WM, white matter; R, right hemisphere*.

Patients with bvFTD compared to HC showed differences in FA values in association fibers such as the ILF (L), IFOF (L), SLF (L), and SLF (R); in projection fibers such as the ATR (R), CST (R), and pontine crossing tract (R), as well as in the right cerebellum, medial lemniscus (R), middle cerebellar peduncle, and WM adjacent to lateral occipital cortex superior division (L), angular gyrus (L/R), temporal occipital fusiform cortex (R), lateral occipital cortex inferior division (R), and pre-central gyrus (R). See Table 7 and Figure 2. No differences were found in FA between EO-OABD and bvFTD.

**Table 7:** Regional brain differences in structural connectivity as measured by FA between HC and bvFTD patients at *p* < 0.01, corrected for multiple comparisons.

**Cluster equivk**	**Peak**	**MNI Coordinates**			**Region with FA differences**
	***p* (unc)**	**x**	**y**	**z**	
1,036	0.002	5	−55	−21	WM cerebellum R
112	0.008	−33	−79	16	Inferior longitudinal fasciculus L, inferior fronto-occipital fasciculus L
92	0.007	−43	−64	18	WM adjacent to the angular gyrus L
89	0.009	14	−30	−27	Anterior thalamic radiation R, medial lemniscus R, middle cerebellar peduncle
84	0.006	42	−48	−18	WM adjacent to angular gyrus R, WM adjacent to temporal occipital fusiform cortex R
77	0.010	41	−74	7	WM adjacent to lateral occipital cortex, inferior division R
73	0.006	9	−21	−29	Corticospinal tract R, pontine crossing tract
65	0.009	36	−8	49	Superior longitudinal fasciculus R, WM adjacent to precentral gyrus R
60	0.008	−24	−59	46	Superior longitudinal fasciculus L

*FA, fractional anisotropy; HC, healthy controls; bvFTD, behavioral variant frontotemporal dementia; MNI, Montreal Neurological Institute; WM, white matter; R, right hemisphere; L, left hemisphere*.

### Correlations Between Clinical Variables and Brain Morphometry

**Figure 3:**
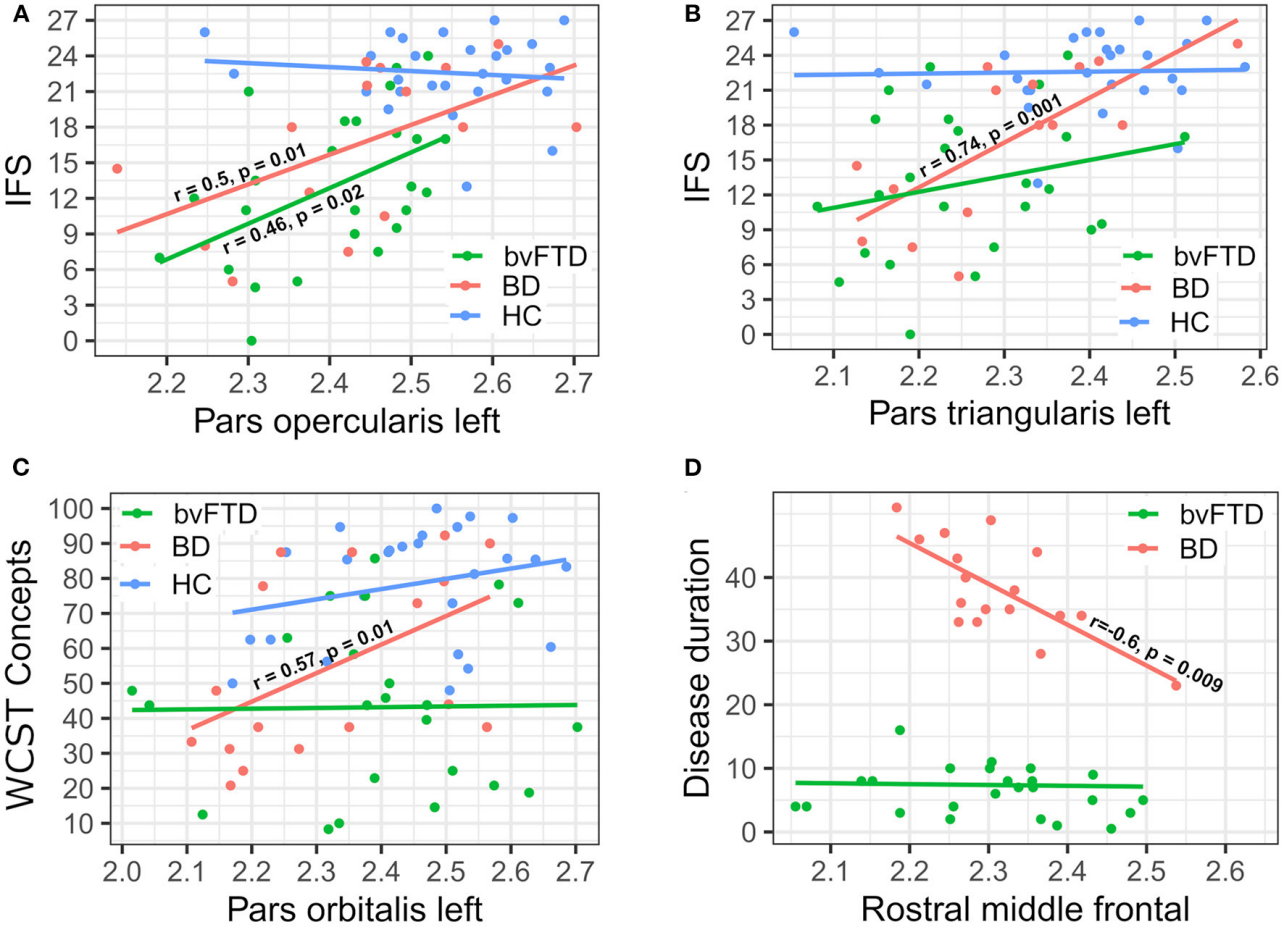
Correlations between cortical thickness and clinical variables. **(A)** IFS vs. Pars opercularis left; **(B)** IFS vs. Pars triangularis left; **(C)** WCST Concepts vs. Pars orbitalis left; and **(D)** disease duration vs. rostral middle frontal. IFS, INECO Frontal Screening; WCST, Wisconsin Card Sorting Test.

Correlations between clinical variables and cortical thickness as measured through SBM showed that the IFS scores were correlated with the left pars opercularis in both EO-OABD (*r* = 0.56, *p* = 0.01) and bvFTD (*r* = 0.46, *p* = 0.02) (Figure 3A), as well as with the left pars triangularis (*r* = 0.74, *p* = 0.001) in the EO-OABD group (Figure 3B). The WCST (conceptualization) correlated with the left pars orbitalis in the EO-OABD group (*r* = 0.57, *p* = 0.01) (Figure 3C). Moreover, only in bvFTD patients, the long-term memory (FCSRT Delayed recall total) was correlated with a decrease in the left entorhinal thickness (*r* = 0.52, *p* = 0.006) and in the left temporal pole (*r* = 0.53, *p* = 0.005). Likewise, intrusion errors were negatively correlated with the right entorhinal (*r* = −0.38, *p* = 0.05), where the more the decrease in cortical thickness, the more the intrusion errors. In EO-OABD and HC groups, no correlations were found between these memory variables and temporal regions (data not shown). Disease duration was correlated with several regions (R cuneus, L rostral middle frontal, L superior temporal, and R temporal pole) in the EO-OABD group (Figure 3D). In contrast, in bvFTD, disease duration was correlated exclusively with the R cuneus.

## Discussion

This study found that most of the cognitive tests and neuroimaging analysis showed significant differences between HC and clinical groups. In contrast, comparisons between EO-OABD and bvFTD showed few differences. EO-OABD and bvFTD patients differed in cognitive measures of *delayed recall* and *intrusion errors* in the memory test and in the variable *WCST conceptualization*. Morphometric analysis showed differences limited to the right temporal pole and the left entorhinal cortex, where the bvFTD group showed lower cortical thickness than EO-OABD. In contrast, the structural connectivity analysis did not show significant differences between EO-OABD and bvFTD. Our results suggest that after a long evolution of a chronic psychiatric disease such as EO-OABD, structural features of gray matter and WM may be affected in regions that may overlap with the areas involved in bvFTD, which may possibly explain similarities in the clinical features observed in both clinical conditions. However, greater alteration in corpus callosum integrity observed in EO-OABD and the compromise in pontocerebellar fibers observed in bvFTD could suggest different regions that are particularly vulnerable in each disease. We discuss the findings in relation to neuropsychological profiles, followed by morphometry and structural connectivity patterns that may constitute similarities as well as differential markers between EO-OABD and bvFTD. Moreover, we will discuss our results in light of previous reports in comparison to HC. Finally, some implications for the differential diagnosis and for further research in the area are discussed.

### Neuropsychological Profiles

The clinical groups (bvFTD vs. EO-OABD) did not differ in several variables belonging to the different cognitive domains evaluated, including *immediate recall* in the memory test, EFs (measured through the *IFS* and most of the *WCST* variables), attentional processes, praxis, and verbal fluency (phonological and semantic). Although the performance of the bvFTD group was lower than the EO-OABD in all variables, except for the *ROCF-time* in which the EO-OABD group showed reduced processing speed than bvFTD, none of these differences reached statistically significant differences. These results are relevant because they suggest that deficits involving multiple cognitive domains may be present in both clinical conditions.

Indeed, both clinical groups showed significantly lower performances than HC in the IFS total score, which is in accordance with previous reports that have documented a significant impairment in EFs in both BD (6, 7, 13, 72, 73) and bvFTD (74–79). The absence of significant differences between EO-OABD and bvFTD in EF measurements may be explained by the extended alterations observed in regions belonging to the PFC in both clinical groups. In fact, we found correlations between IFS and cortical thickness of the left pars opercularis in both EO-OABD and bvFTD groups. Other regions, such as the left pars triangularis and the left pars orbitalis, were correlated with the IFS only in the EO-OABD group. The last result may suggest that in the EO-OABD group, decreased performance in EF seems to be closely related to focal atrophy in the frontal regions. The clinical groups only differ in an EF variable related to cognitive flexibility (*WCST conceptualization*). The WCST is considered a highly sensitive tool to evaluate EFs and may involve complex thought processes, being considered a specially demanding test that recruits diverse cognitive components and several neural correlates, including not only regions typically associated to EFs such as the DLPFC but also regions as the right posterior cingulate and cerebellar regions (80). The bvFTD group showed alterations in gray matter of the left posterior cingulate and in WM at the level of the cerebellum that were not present in the EO-OABD group. It could suggest a more widespread structural compromise that may influence diverse cognitive components, possibly explaining the major sensitivity of the WCST to detect differences between EO-OABD and bvFTD.

Regarding the memory domain, the clinical groups did not differ in terms of *immediate recall* (free and cued), while in *delayed recall* trials the EO-OABD group showed better performances than bvFTD. Moreover, the number of intrusions was significantly higher only in the bvFTD group, suggesting that the inhibitory mechanisms required to suppress unrelated responses during recall in memory tests may be particularly altered in this clinical condition. On the other hand, although EO-OABD patients showed lower performances than HC in all memory measurements, the higher scores obtained in *delayed recall* trials in comparison to bvFTD and the absence of significant intrusion errors suggest that the alteration in memory processes tends to be milder in EO-OABD than in bvFTD. This is the first time that EO-OABD and bvFTD are compared regarding memory processes by which no previous results can be discussed. Nonetheless, it is relevant to consider that in comparison to HC, in bvFTD the alteration in memory processes has been typically described as predominant in retrieval processes, while the storage of new information is described as relatively preserved (78, 81). However, our results suggest that bvFTD patients may present failures in both storage and retrieval processes as reflected by significantly low performances in immediate and delayed recall in both free and cued trials. Moreover, only in the bvFTD group, *intrusion errors* were negatively correlated with the right entorhinal, while *delayed recall* scores were correlated with the left entorhinal and the left temporal pole, which may suggest that the memory profile may be more relevant as a marker of neuropsychological dysfunction in bvFTD than in EO-OABD, probably due to the more widespread alteration of temporal regions observed in the bvFTD group.

Generally, one of the most altered processes in BD is attention (6, 7, 13, 82); we consistently found low scores in structured tests (SDMT) and a decrease in processing speed in the EO-OABD group. We also found disturbances in praxis and in the phonological and semantic verbal fluency, even when deficits in these cognitive domains are not generally described as part of the cognitive impairment profile in BD (6) and bvFTD (74, 79). Although these results suggest a compromise in multiple cognitive functions, it is relevant to consider that wide variability in the distribution of cognitive performance was observed in both clinical groups, where some patients obtained extremely low scores, while others showed performances within the expected range. The variability in cognitive performance in bvFTD could be related to disease duration. The initial symptoms of bvFTD involve mainly the behavioral component, while cognitive impairment often appears after disease progression (77, 83, 84). In the present study, disease duration in the bvFTD group ranged from 1 to 16 years, which could explain the variability in cognitive profiles. In BD, neurocognitive alterations seem to be related to multiple factors, such as pharmacological treatments, comorbidities with other psychiatric disorders and cardiovascular disease, and particularly the number of prior episodes (85). However, other studies have not found a clear association between cognitive performance and episode recurrence (86). Our effort to objectively establish the number of episodes was not enough to obtain precise information. Due to the long disease duration, this information tended to be very imprecise, due to which we were not able to explore the correlation between clinical and neuroimaging variables and the number of mood episodes in our EO-OABD group.

### Morphometric Profiles

Although in comparison to HC cortical surface reduction was more evident in bvFTD than in EO-OABD, comparisons performed within the two clinical groups showed significant differences only in the right temporal pole and the left entorhinal cortex, in which the bvFTD group showed reduced cortical surface. The clinical relevance of these differences remains to be elucidated. Several studies have associated neurodegenerative disorders belonging to the FTLD spectrum with focal alterations in the temporal pole, a complex region related to a broad quantity of cognitive processes, including visual processing for complex objects, face recognition, autobiographic memory, naming, and word-object labeling, as well as semantic processing in all modalities and socio-emotional processing (87, 88). On the other hand, the entorhinal cortex has been associated with memory consolidation thanks to its connection with the medial prefrontal/anterior cingulate cortex (89). A deeper damage in the left entorhinal cortex observed in our bvFTD group, compared to EO-OABD, may explain significant differences that also emerged in memory variables, as well as the fact that correlations between the left entorhinal cortex and *delayed recall* were found to be significant only in the bvFTD group, as previously discussed.

Similarities between bvFTD and EO-OABD point to the reduced cortical surface that both clinical groups showed when compared to HC involving bilateral regions on the rostral middle frontal and superior frontal cortex, as well as in the right hemisphere at the level of caudal middle frontal, pars opercularis, pars triangularis, superior temporal, pre-central, and superior parietal. On the other hand, only the bvFTD group showed decreased surface compared to HC in the left superior parietal, posterior cingulate, isthmus cingulate, fusiform, lingual, and parahippocampal as well as in the right post-central, precuneus, pars orbitalis, and inferior parietal. Likewise, only the EO-OABD group showed reduced surface area when compared to HC in the bilateral frontal pole, cuneus, and transverse temporal, in the left pre-central, paracentral, pars opercularis, pars triangularis, pars orbitalis, caudal middle frontal, and superior temporal, as well as in the right supramarginal, lateral occipital, middle temporal, and inferior lingual. Nonetheless, these regions did not show significant differences when comparisons were performed between the clinical groups. Only two studies have compared brain morphometry in BD and bvFTD patients before (38, 39), finding that brain changes in elderly BD patients were not as severe as those observed in bvFTD (42) and that PFC gray matter reduction showed different localization between groups, with a greater reduction in DLPFC in bvFTD and predominant reduction in VLPFC in BD (43). Although we also found that BD patients exhibited less atrophy than bvFTD when compared to HC, comparisons between BD and bvFTD showed few differences focused exclusively on temporal regions. One explanation for the few differences that emerged between our EO-OABD and bvFTD groups may be related to the disease duration. In the previous studies, the disease duration was described as more than 10 years (42) and 14.6 ± 7.2 years (43), respectively. In our study, the EO-OABD group had a disease duration of 38 ± 8 (range: 23–51) years and an age range of 54–81 years old. Thus, it is possible to consider that when BD patients are evaluated at an older age or after a long time of disease progression, they may exhibit deeper structural changes, more closely related to those observed in bvFTD. Indeed, we found that disease duration was correlated with the cortical surface in several regions, including the right cuneus and temporal pole, as well as the left rostral middle frontal and superior temporal, exclusively in the EO-OABD group (Figure 3D), while in bvFTD, disease duration was only correlated with the right cuneus. These results suggest that in EO-OABD patients, the longer disease duration may be related to a more significant loss of gray matter, which predominantly involves cortical regions of the frontal and temporal lobes.

Considering the results obtained by each group when compared to HC, our results are consistent with previous reports showing differences in cortical morphometry in BD patients involving the frontal, temporal, and parietal regions (90–92), in which the recurrence of mood episodes also seems to be related to alterations of cortical morphometry (93). Although we could not evaluate correlations between brain morphometry and the number of mood episodes, correlations found with the disease duration may confirm some associations between disease progression and cortical damage in BD. On the other hand, in bvFTD gray matter decrease in the frontal and anterior temporal lobes, involving mainly the orbitofrontal gyrus and the insula, have been reported (50, 94). We have found that bvFTD patients showed a significant reduction in cortical surface area in regions belonging to the frontal, temporal, parietal, and occipital lobes, suggesting extensive cortical damage in this clinical group. Our results are consistent with the dynamic nature of the phenotypes observed in FTD over long periods (44), highlighting that although the early structural changes in bvFTD are relatively focal, disease evolution conduces to a progressive alteration in posterior brain regions.

### Structural Connectivity Profiles

In this section, first, we will discuss the tracts in which FA was significantly decreased in both BD and bvFTD in comparison to HC. Second, we will examine the tracts that showed FA reduction exclusively in BD when compared to HC and, finally, the tracts that showed FA reduction exclusively in bvFTD when compared to HC, discriminating by association, projection, and commissural fibers. Since no previous studies have been performed regarding comparisons of structural connectivity patterns between EO-OABD and bvFTD, we will discuss our results in light of the literature that involves comparisons with HC.

#### Structural Connectivity Differences in Both BD and bvFTD When Compared to HC

In comparison to HC, both bvFTD and EO-OABD patients showed FA differences in association fibers such as the bilateral SLF, the left ILF, and the left IFOF. These tracts have been previously reported to be disrupted in both BD (45, 95) and bvFTD (47, 48, 96); consequently, some insights about their function are briefly reviewed here. The SLF connects the frontal, occipital, parietal, and temporal lobes and constitutes a key connectivity structure of the cognitive control network (CCN), a network associated with attentional and executive processes (97). Moreover, this tract has been associated with emotional regulation and language processing (95), and its disruption is thought to contribute to a frontotemporal disconnection that could be involved in emotional modulation and inhibition alterations observed in BD (45) and bvFTD (98). The ILF connects the occipital lobe with the anterior part of the temporal lobe, and it is associated with language and emotional evaluative processes, as well as with visual processing of verbal information (99–101). The IFOF connects the inferior lateral prefrontal cortex and DLPFC with posterior temporal and occipital cortices and has been involved in many brain functions, particularly in inhibitory control and cognitive flexibility in BD (95), as well as in behavioral markers related to apathy in bvFTD (98). These tracts seem to be highly relevant in processes that involve cognitive, behavioral, and emotional components; thus, their disruption may explain the clinical features shared by both clinical groups.

The clinical groups also differ from HC regarding projection fibers such as the right ATR and the CST. The ATR connects the PFC (mainly DLPFC) and the dorsomedial thalamic nucleus through the anterior limb of the internal capsule and functionally is involved in EFs and complex behaviors (102). The ATR has been reported to be altered in bvFTD (47, 48, 96) and in BD patients (51, 102, 103), in which it has been associated with performances in attention, information processing, and working memory (95). On the other hand, the CST is part of the descending motor pathway and is involved in the execution of discrete voluntary movements (104). Only one study has reported alterations in the CST in BD, and the authors have proposed that its alteration could be related to failures in motor skills and also to serotonin and mood regulation (99). Although no direct evidence of CST compromise has been reported in bvFTD, syndromes belonging to the FTLD spectrum have been related to alterations in central motor conduction and structural changes in the CST (105, 106), particularly in patients with TDP-43 type C pathology (107, 108), supporting some hypotheses about altered motor system function in FTLD (105). A considerable proportion of FTLD patients, including bvFTD, may be more at risk of motor system dysfunction than the general population (49, 109), and progression to a diagnosis of motoneuron disease is higher among bvFTD patients (110); therefore, it cannot be ruled out that some of our patients could fulfill criteria for motoneuron disease, given the long history of disease in some patients and the presence of motor function alterations reported in 32% of our bvFTD group.

Finally, WM disruption in the right cerebellum was found in our group of EO-OABD and bvFTD patients when compared to HC. Some findings on the implication of the cerebellum have been previously reported in both BD (111, 112) and bvFTD (113, 114). Beyond the motor function of the cerebellum, it has also been involved in cognitive processes (115), with evidence of its participation in social cognition in bvFTD (114, 116), as well as in mood regulation components in BD (51).

#### Structural Connectivity Differences Found Exclusively in BD

Compared to HC, patients with BD showed FA differences in commissural fibers such as the bilateral body of corpus callosum and the forceps minor and major, in association fibers such as the left uncinate fasciculus and the bilateral cingulum, and in projection fibers of the PTR. Disruptions in the corpus callosum and forceps minor and major have been broadly reported in BD (45, 117, 118), due to which alterations in the interhemispheric communication have been suggested as a relevant phenotype in this psychiatric condition (119, 120). This neuroanatomical marker suggests the relevance of exploring in BD patients some clinical features of the callosal syndrome, also named split-brain, such as the extinction of functional integration of perceptual information, which involves surprising alterations in consciousness processes (121). These symptoms have been broadly described in patients with corpus callosum ablation due to epilepsy (122). But, so far, no reports have been found in BD patients. Thus, further studies are needed to evaluate the clinical implications of disruptions in interhemispheric communication in BD, considering the integration of perceptual information, the integration between emotions and language, and more complex processes related to the conscious experience. Association fibers such as the uncinate fasciculus and the cingulum belong to the fronto-limbic network. The uncinate fasciculus connects limbic areas such as the amygdala and hippocampus to frontal regions (97), while the cingulum collects projections from the cingulate gyrus and reaches the amygdala passing around the ventral surface of the hippocampus (123). The role of this network in emotional information processing, as well as its disruption in mood disorders, has been consistently demonstrated (97, 118); thus, it is not surprising to find alterations in these tracts in elderly patients with an EOBD. From a perspective of treatment, this network is highly relevant since WM underlying the subgenual anterior cingulate cortex (sACC) has been identified as a target of intervention in mood disorders. The sACC (Brodmann area 25) is a subregion of the subcallosal cingulate (SCC), identified as the intersection of forceps minor, the uncinate fasciculus, the cingulum, and fronto-striatal fiber bundles (124). Deep brain stimulation of the sACC has demonstrated a striking improvement of treatment-resistant depression (125), which may be mediated *via* strong connections to the orbitofrontal cortex (OFC), anterior midcingulate cortex (AMCC), hypothalamus, nucleus accumbens, amygdala, and hippocampus (126). Although the causal relation between the sACC and mood symptomatology is not completely clear, the mean gray matter volume of the sACC has been reported to be abnormally reduced in both subjects with major depressive disorder (MDD) and subjects with BD, irrespective of mood state. Likewise, metabolism appeared to be increased in sACC in mood disorders (after correction for volume differences) (127). Therefore, our results regarding the alteration of WM surrounding subcallosal regions, and the studies described, highlight the relevance of continuously exploring deep brain stimulation therapies using sACC as a target of intervention in BD, which has demonstrated a potential efficacy in BD comparable to that obtained in patients with MDD (128). Moreover, it remains to be elucidated whether deep brain stimulation could represent a protective factor for a better prognosis in OABD due to its potential capability to reduce recurrence of mood episodes.

#### Structural Connectivity Differences Found Exclusively in bvFTD

In the bvFTD group, when compared to HC, FA reduction was found in the right middle cerebellar peduncle and the pontine crossing tract, as well as in WM adjacent to the right angular gyrus, the right temporal occipital fusiform cortex, and the right pre-central gyrus. The middle cerebellar peduncle fibers connect the contralateral pontine nuclei to the opposite hemisphere of the cerebellar cortex (115). Although markers of disruption in the middle cerebellar peduncle have not yet been reported in bvFTD, changes in cerebellar function and structure may be of particular clinical relevance in this disease (116). No other major tracts showed significant differences in bvFTD when compared to HC.

### Implications of the Results

It has been suggested that “although the lifelong BD may go onto develop bvFTD, it is late-onset BD that carries the most significant risk for developing bvFTD” (30); however, the present study has been focused precisely on those patients with “lifelong BD,” thus including only patients classified as EO-OABD. Our approach allows us to characterize BD patients after a long progression of the disease and is motivated by the fact that EO-OABD patients constitute a population that will continue to increase. They are considered as a “*healthy survivor BD sub-population*” given the high mortality of the disease (21). Thereby, studies focused on this population may better characterize a disease that has been suggested as “neuroprogressive” to explore whether it courses with features similar to those observed in neurodegenerative diseases. This approach may be especially relevant for neurologists, geriatrists, and neuropsychologists who will continue to evaluate elderly patients with chronic psychiatric disorders complaining of cognitive disturbances. Also, it may be appropriate for psychiatrists who will observe different aging courses in their patients and who will require to identify the patients that may present pathological aging.

We have focused our study on comparing EO-OABD with bvFTD due to the intriguing associations with this specific type of dementia. Several ways of approaching the question of a relationship between BD and FTD were synthesized by Papazacharias et al. (23), including: “(1) *sharing pre*-*disposing factors*, mainly genetics, (2) *causal relationship* in which BD patients are at greater risk for developing FTD, (3) *reverse relationship* in which FTD presents with a bipolar-like syndrome, (4) *sporadic co-occurrence* of BD and FTD, (5) *late-onset BD preceding the diagnosis of FTD*, or (6) *specific dementia syndrome arising as a result of bipolarity* but that does not seem to correspond to the criteria of the main types of dementia, including FTD.” Possibly all these cases can be found in clinical practice. Among the patients evaluated in our sample of EO-OABD, we have identified three patients (17%) to whom, after careful evaluation in our memory clinic, a diagnosis of dementia was suspected. Nonetheless, the differential diagnosis was challenging, and the possible dementia was associated with the baseline psychiatric disease. Moreover, three patients (17%) were classified as having normal cognition. In contrast, the remaining patients (*n* = 11, 64%) were classified as having a mild deficit in cognitive processes, requiring further longitudinal evaluations to discard progression. In general, studies evaluating cognition in BD tend to exclude patients with dementia (129); however, it can lead to a selection bias that does not allow characterizing a complete profile of OABD patients.

The evolution of BD is not easily predictable, and its pathophysiological mechanisms are not yet fully understood. For example, the etiology of progressive impairment in BD may involve injury due to neuroinflammatory activity and oxidative stress, among other pathophysiological changes related to accelerated aging (1, 82), glial loss (46), the aggregation of vascular disease (130), sleep and circadian disruptions (131), and pharmacotherapy, since the use of lithium or anticonvulsants may confer various risks for dementia (132). Whatever the etiology of the progression to dementia in BD, this seems to be an outcome that cannot be generalized to all patients. We have identified risk factors such as hypertension, diabetes mellitus, and alcohol consumption in our EO-OABD sample. However, the reduced sample size was a limitation to conduct regression analyses focused on evaluating the impact of these variables and pharmacological treatments on the cognitive and neuroimaging profiles.

Since 64% of our sample was classified as having a mild cognitive deficit without evidence of dementia, we could consider that this group may represent a population of patients with a *mild neurocognitive disorder associated with EO-OABD*. These patients may not necessarily evolve to dementia but may present cognitive deficits, behavioral changes, and neuroimaging features that may mimic a non-progressive bvFTD syndrome (bvFTD phenocopy). Longitudinal studies in EO-OABD, including patients with normal cognition, patients with mild cognitive impairment, and patients with dementia, are specially required to characterize the progression in these different groups and to identify the factors that may improve the prognosis of the disease.

On the opposite direction, associations between bvFTD and previous psychiatric diagnosis are equally complex. The retrospective study of Mendez et al. (30) revealed that 10.2% of bvFTD patients had a previous diagnosis of BD; a deeper analysis of their histories confirmed a BD diagnosis in 11 patients (8%), among whom 3 patients (2.1%) had non-progressive bvFTD while the remaining 8 patients (5.8%) fulfilled the criteria for progressive bvFTD, concluding that the relationship between bvFTD and BD may be rather heterogeneous (30). We did not identify a previous history of BD in our bvFTD sample. Only one patient reported an episode of depression occurring in response to a stressful event several years before the onset of bvFTD, which was not related to the actual disease. The possibility of discriminating between bvFTD and primary psychiatric disorders is relevant in cases of late-onset behavior changes. In this context, the study of Vijverberg (133) identified that variables such as gender, stereotypy, depressive symptoms, and neuroimaging contribute to the differential diagnosis. However, they also found that 33% of patients diagnosed with bvFTD demonstrate depressive symptoms (133). Similarly, among our bvFTD sample, depressive symptoms were reported in some patients (*n* = 5, 20%) in the course of the actual disease, reiterating the complexity of the interaction between mood disturbances and the bvFTD syndrome.

From our results, the absence of differences in structural connectivity profiles and the scarce differences regarding SBM and neuropsychological profiles that were found in the comparisons between EO-OABD and bvFTD may suggest the existence of common underlying mechanisms between both clinical conditions, even when the etiology of each disease can be different and multifactorial. One of the hypothetical mechanisms could involve the functional correlate typically associated with the pathophysiology of BD: the alteration in prefrontal-limbic connectivity whereby the prefrontal regions fail to regulate limbic regions leading to the emotional instability characteristic of the disorder (36, 45). It is possible that with the disease progression and the accumulation of excitotoxic processes, this functional correlate may lead to structural alterations (42). Consequently, progressive damage in regions that are vulnerable from the early stages of BD could lead to anatomical changes that may overlap with regions altered in syndromes belonging to the FTLD spectrum, leading to a neurocognitive disorder associated with EO-OABD that may mimic bvFTD syndrome, being non-progressive (as the phenocopy syndrome) or progressive depending on the chronicity of the disease and the particular accumulation of risk factors for dementia.

### Future Directions

Further studies are required to continue to understand the interaction between FTD and BD. Since not all EO-OABD cases evolve to dementia, significant efforts must continue to be made to identify protective factors that may contribute to a better prognosis of the disease. Long-term longitudinal designs—including EO-OABD patients with different profiles: normal cognition, mild cognitive impairment, and dementia—are specially required to characterize the progression in these groups. A complete characterization of the pharmacological treatment and the level of adherence to the treatment must be performed to understand the effect of episode recurrence and pharmacotherapy on cognitive and neuroimaging outcomes in EO-OABD patients. Our findings have shown that some cognitive domains—particularly memory—seem to be more characteristically altered in bvFTD than in EO-OABD. Thus, further studies including a complete neuropsychological battery evaluating all cognitive domains, including memory and complex EFs such as cognitive flexibility, are recommended to explore differential cognitive markers between EO-OABD and bvFTD.

Since in our analyses of structural connectivity we have found that the corpus callosum was the most prominently affected fiber in the EO-OABD group, confirming the interhemispheric connectivity disruption as a trait marker of the disease (134) and that WM disruptions in ponto-cerebellar areas were particularly prominent in the bvFTD group and suggesting an implication of alterations in the motor system in this clinical condition (49, 109), structural connectivity patterns may also contribute to identifying differential markers between these clinical conditions. A familial history of psychiatric disease may also constitute a differential marker since in our sample a positive familial history of psychiatric disease was significantly more prevalent in EO-OABD patients (94%) than in bvFTD (32%). Finally, since differences in neuroimaging profiles for the identified genetic mutation (C9orf72 vs. GRN) have been identified in bvFTD (135, 136), further studies should also consider genetic risk variants in relation to neuroanatomical and clinical features that converge between EO-OABD and bvFTD, which may allow deepening our understanding of their shared underlying mechanisms.

### Limitations

One of the limitations of our study is the cross-sectional design, due to which no conclusions can be drawn about the progression of BD. Another limitation was the difficulty in obtaining precise information about the number of mood episodes. Future studies, preferably with a longitudinal design that allows a careful characterization of the sample, must be conducted to explore the impact of the number of episodes and pharmacological treatments on the neuroimaging and clinical profiles observed in elderly BD patients. The setting for the recruitment of patients, a memory clinic, may have increased the probability of enrolling BD patients with cognitive impairment; therefore, future studies must recruit patients through public calls or directly in psychiatric units to obtain a greater heterogeneity of clinical profiles. A selection bias must be considered for the bvFTD diagnosis due to the complexity of a differential diagnosis in neurodegenerative diseases and the requirement of neuropathological markers as the final confirmatory test to obtain a definitive diagnosis. None of the cases had post-mortem confirmation after completion of the study. Likewise, fluorodeoxyglucose (FDG)-PET was not used to confirm the diagnosis. However, we counteract the risk of selection bias through an interdisciplinary evaluation and a diagnosis established through expert consensus, following the diagnostic criteria of Rascovsky et al. (53), based on clinical information and supported by structural MRI images. Finally, sample size also constitutes a limitation of this study. Although it was attempted to include a control group that was comparable in demographic features to the clinical groups, the results derived from this study must be interpreted in the context of a descriptive and exploratory approach that may guide hypotheses for further research in the field.

## Data Availability Statement

The datasets presented in this article are not readily available because raw data from patients are only available in institutional repositories. Requests to access the datasets should be directed to dianamat@javeriana.edu.co.

## Ethics Statement

The studies involving human participants were reviewed and approved by Ethics Committee of the Hospital Universitario San Ignacio/Pontificia Universidad Javeriana. The patients/participants provided their written informed consent to participate in this study.

## Author Contributions

FC-S, DM, and PR designed the study and implemented the research. FC-S, PR, MG-G, and CT-M contributed to the analysis of the results and to the writing of the manuscript. CC, RP, and DM performed a critical revision of the article. All authors contributed to the article and approved the submitted version.

## Conflict of Interest

The authors declare that the research was conducted in the absence of any commercial or financial relationships that could be construed as a potential conflict of interest.

## Publisher's Note

All claims expressed in this article are solely those of the authors and do not necessarily represent those of their affiliated organizations, or those of the publisher, the editors and the reviewers. Any product that may be evaluated in this article, or claim that may be made by its manufacturer, is not guaranteed or endorsed by the publisher.
